# Work-Related Burdens and Requirements for Health Promotion Programs for Nursing Staff in Different Care Settings: A Cross-Sectional Study

**DOI:** 10.3390/ijerph16193586

**Published:** 2019-09-25

**Authors:** Ann-Kathrin Otto, Laura L. Bischoff, Bettina Wollesen

**Affiliations:** Institute of Human Movement Science, University of Hamburg, 20148 Hamburg, Germany; laura.bischoff@uni-hamburg.de (L.L.B.); bettina.wollesen@uni-hamburg.de (B.W.)

**Keywords:** nursing staff, work-related burdens, requirements, health promotion programs

## Abstract

Nursing staff in all settings have multiple work-related problems due to patient handling and occupational stressors, which result in high stress levels and low back pain. In this context the importance of health promotion becomes apparent. The aim of this study is to analyse whether nursing staff (in elderly care, hospitals, home care, or trainees) show different levels of work-related burdens and whether they require individualized components in health promotion programs. N = 242 German nurses were included in a quantitative survey (Health survey, Screening Scale (SSCS) of Trier Inventory for Chronic Stress, Slesina). The differences were tested using Chi^2^-Tests, Kruskal–Wallis Test and one-way ANOVA. Nurses differed in stress loads and were chronically stressed (F_(3236)_ = 5.775, *p* = 0.001). Nurses in home care showed the highest SSCS-values with time pressure as the most important straining factor. The physical strains also placed a particular burden on nurses in home care, whereas they still reported higher physical well-being in contrast to nurses in elderly care (Chi^2^ = 24.734, *p* < 0.001). Nurses in elderly care and home care preferred strength training whereas nurses in hospitals and trainees favoured endurance training. Targeted programs are desirable for the reduction of work-related burdens. While nurses in elderly care and home care need a combination of ergonomic and strength training, all nurses require additional stress management. Planning should take into account barriers like perceived additional time consumption. Therefore, health promotion programs for all settings should be implemented during working time at the work setting and should consider the working schedule.

## 1. Introduction

In 2018, about 1.6 million people were employed in nursing settings in Germany [1]. At the same time there are difficulties recruiting and retaining staff. As a result, there is a shortage of nurses in the labor market [2,3]. Subsequently, this leads to high levels of work-related strain and stress among nursing staff.

High workloads, time pressure, lack of task control, role ambiguity, inadequate staffing levels, low income, and workplace violence have been identified as nursing specific workplace stressors [4,5,6,7,8]. Especially burnout levels are high [9] and may even be higher than those of other professions [10,11]. Even though many of the discussed occupational influences of nurses and trainees are similar in different settings, such as stationary or ambulant and hospital or elderly care there are some particular stressors in each setting.

Nursing staff in elderly care experience higher physical and emotional strain than nursing staff in home care [12,13], whereas, nursing staff in home care show lower values for quantitative and emotional demands and less work-privacy conflicts [13]. Moreover, the latter show higher ratings for job insecurity. When compared to employees in hospitals, nursing staff in elderly care and home care showed more positive values in almost all aspects of their working situation [13]. In contrast, nursing trainees claim to be more dissatisfied with their professional reputation and feel more emotionally burdened than nursing staff due to their limited professional influence [14]. In addition, nursing trainees find it stressful to apply their theoretical classroom learnings in the field while pressured with the sense of urgency to help people in distress [15].

In comparison to other professions, nursing staff have a six times higher prevalence of back injury [16]. Frequent physical tasks such as heavy lifting and transferring, particularly in awkward body positions due to time pressure, could lead to lumbar disc narrowing and tissue damage, which can subsequently result in lumbar spine disease and low back pain [17,18]. Moreover, psychosocial factors like high demands, low decision latitude, lack of appreciation and recognition were significantly associated with low back disorders [19,20]. Nursing staff in elderly care have the highest lifting and bending exposure (63.5) with an increased risk of disability in comparison to nursing staff in hospitals (53.2) and home care (34.8) [20]. For example, the NEXT study analyzed the disabilities of 21,516 health care workers from 11 countries using a four-item-scale by Korff et al. [21]. It considered neck and/or low back pain in the past six months and the interference with social and daily activities as well as the ability to work. This indicates that the percentage of nursing staff with disability, due to neck/shoulder and low back pain, is highest in elderly care (48%) in comparison to nursing staff in hospitals (46.2%) and home care (37.8%) [20]. 

High numbers of sickness absences among nursing staff are a result of these conditions [22,23]. There are no significant differences with respect to duration of sick leave between nursing staff in elderly care and home care [24]. Quite contrary, in 2017 there was an average of 23.8 sick-leave days documented for German nursing staff in elderly care whereas there was only an average of 18.2 sick-leave days documented for hospital nursing staff [25]. These differences must be a result of the handling of residents with more disabilities [26] along with higher occurrences of psychosocial stressors in elderly care such as resident aggressions [27] and death [28]. Moreover, increasing workloads—due to a higher percentage of old people with increasingly complex health needs—and the shortage of staff lead to significant work intensification in elderly care. Conversely, nursing trainees showed less lifting and bending exposure [29] and consequently less musculoskeletal disorders in comparison to registered nurses [30].Nevertheless, it seems necessary to address trainees’ health competencies.

With regard to the high levels of work-related burdens among nursing staff the importance for occupational health promotion becomes apparent.

Longitudinal studies evaluating the effects of health promotion programs in nursing staff are increasingly emerging [31,32]. A systematic review [16] assessed the effect of different interventions that aim to prevent back pain and back injury in nurses of all nursing settings. Moderate evidence was found regarding multidimensional manual handling training strategies and even conflicting evidence for the efficiency of exercise interventions was gathered. Systematic reviews regarding the effect of interventions on stress in nursing personnel [33,34] show similar results. Only a few studies show a tendency towards a protective effect of physical activity on stress in healthcare workers [35,36,37]. Nevertheless, Edwards et al. [33] conclude that the sources of stress at work in health care and its impact on a range of outcome indicators are well researched, but a translation of these results into well evaluated interventions is lacking. Small sample sizes, low adherence, and high dropout rates are points of contention which may be due to setting specific factors like staff shortages, shift work, organizational barriers, and time pressure [34,38]. Reasons for methodological problems in intervention research are diverse and are exceptionally difficult to avoid in the health care setting. This further highlights the need for participant-tailored interventions that take into account the target populations’ wishes, needs and barriers [34]. With earlier studies in mind that substantiated different strains and stressors in other nursing settings, these wishes, needs and barriers for tailored health promotion programs might differ among the different nursing roles. Nursing staff is generally motivated to participate in health promotion programs but several studies have also shown that the practical nursing tasks are prioritized over health promotion [39,40].

Therefore, the aim of this study is to analyze whether nursing staff in different roles (nursing staff in elderly care, in hospitals, in home care, or trainees) show different levels of work-related burdens and whether they have particular and individualized requests and barriers regarding participation in exercise and health promotion programs. To conduct tailored interventions in the future, the research questions are the following:

1. What are the differences of nurses in different roles regarding psychological strains (e.g., Mental Health, Chronic Stress, Mental Strains)?
2. What are the differences of nurses in different roles regarding physiological strains (e.g., Physical Health, Physical Strains)?
3. What are the differences of nurses in different roles regarding requests and barriers for participation in health promotion programs, especially in physical activity?

We hypothesized significant differences between nursing staff in different roles in terms of physiological strains as well as different requests and barriers in health promotion programs.

## 2. Materials and Methods 

### 2.1. Study Design

This quantitative study was conducted in nursing home facilities, hospitals, and home care services in the German states Hamburg and Baden Württemberg between January 2017 and June 2018. German nursing staff from the third year as an apprentice onward were invited to complete the anonymous survey. Participation in the survey was optional and subjects gave their informed consent. This study followed the ethical standards of the declaration of Helsinki.

### 2.2. Participants

In order to reach a high number of nurses, the health care facilities were contacted through various information channels (telephone calls, personal letters and care networks). The questionnaire was advertised and distributed at information events and sent online. Initially, 539 nurses were invited for participation in the study. N = 242 nursing staff, aged between 17–64 years, completed the standardized questionnaire. Nursing staff were included in the questionnaire study if they were active in stationary or home care or apprentices from the third year onward. Other employees like kitchen staff, home management, professional housekeepers, and psycho-social carers were excluded. This survey had a response rate of 45%. The participants were classified in four groups: 142 nursing staff in elderly care (mean age = 40.70 ± 12.22), 44 nursing staff in hospitals (mean age = 29.45 ± 11.16 years of age), 20 nursing staff in home care (mean age = 30.20 ± 11.17 years of age) as well as 36 nursing trainees (mean age = 25.33 ± 7.89 years of age). The percentage of included men was ≤ 10%. 

### 2.3. Outcome Measures

The questionnaire was generated based on a literature analysis. The survey was subdivided in three sections: (1) Three standardized instruments, (2) requests and barriers for an individualized intervention, (3) demographic data and level of physical activity. The following standardized questionnaires were used in the German version:

#### 2.3.1. Health Survey (SF 12)

The Health Survey (SF12) is an economic short form of the SF-36, consisting of twelve items, to measure the health-related quality of life. Physical and Mental Health Summary Scales (PCS-12 and MCS-12) were used to measure the participants Physical and Mental Health Status [41]. The Summary Measures were each derived from four subscales: Physical Health (PCS) consists of Physical Functioning (PF), Role-Physical (PR), Bodily Pain (BP) and General Health (GH) whereas Mental Health (MCS) is summarizing the subscales Vitality (VT), Social Functioning (SF), Role-Emotional (RE), and Mental Health (MH). They were asked e.g.: “How would you describe your state of health in general?” (answer options: “excellent”, “very good”, “good”, “fair”, and “poor”) or “To what extent have you been hampered in the past four weeks in performing your daily activities at home and at work?” (answer options: “not at all”, “a little bit”, “moderately”, “quite a bit”, and “extremely”). The Scales reliability and validity are considered established. Cronbachs Alpha ranged between 0.57 to 0.94 [42]. The German version of the SF-12 was translated and validated in accordance with the standards of the International Quality of Life Assessment Group [43].

#### 2.3.2. Trier Inventory for Chronic Stress (TICS)

The twelve-item Screening Subscale (SSCS) of the Trier Inventory for Chronic Stress (TICS) was developed by Schulz, Schlotz and Becker and provides information about perceived stress within the last three month [44]. The Screening Scale of the TICS (SSCS) is composed of 12 items and covered five aspects of stress, including lack of social recognition, chronic worrying, work-related and social overload and excessive demands. Participants can rate e.g.: “Times when I worry a lot and cannot stop”, “Fear of something unpleasant happening”. Stress frequency are rated on a four-point scale (0 = never, 1 = rarely, 2 = sometimes, 3 = often, 4 = very often). Internal consistency (Cronbachs Alpha) with a range from 0.84 to 0.91 indicates good to very good reliability. The Trier Inventory of Chronic Stress was translated and validated in German version [45]. 

#### 2.3.3. Questionnaire for Subjective Assessment of Workplace Exposure (modified Slesina Questionnaire)

The frequency as well as the perceived strain of work-related demands was assessed by 15 self-developed items (physical, psychological, and environmental factors). Participants were asked whether a specific demand occurred “frequently” or “seldom” and whether that demand felt physically straining (“yes” or “no”). These 15 items included heavy physical tasks, awkward postures, overhead working, holding heavy load, carrying heavy load, lifting heavy load, awkward static postures, the lack of physical activity, sitting, pressure to perform, deadline pressure, time pressure, visual detail distinction, poor illumination, and IT problems (items translated into English) [46].

#### 2.3.4. Requests for Participation in an Individualized Intervention 

The participants were able to state preferences regarding health promotion programs. Examples of self-developed items are: “Would you like to participate in a health promotion program?” (“yes” or “no”) “What kind of exercise promotion program would you be interested in?” (answer options included strength training, endurance training, and relaxation training).

#### 2.3.5. Barriers to Participate in Health Promotion Programs

Participants were also able to state barriers preventing them from participating in health promotion programs. They were asked the following: “If you currently are not attending any health promotion program, please mark with a cross the barriers that are possibly keeping you from doing so” (answer options: e.g., “I feel too strained”, “additional activities are too time consuming”, “programs are located too far away”, “I don’t know of any suitable offers”, “I don’t want to play sports with my colleagues”).

#### 2.3.6. Physical Activity 

The level of physical activity was assessed via self-developed questions according to WHO criteria for physical activity regarding the duration of weekly and daily activity, e.g., “How many hours per week do you spend exercising/playing sports?” (answer options: “more than 4 h”, “3.5–4 h”, “2.5–3 h”, “1–2 h”, “less than 1 h”) or “How many minutes per day do you walk or ride a bike?” (answer options: “more than 45 min”, “30–45 min”, “15–30 min”, “5–15 min”, “less than 5 min”) (items translated into English).

It took approximately 30 min to complete the entire questionnaire.

### 2.4. Data Analysis

Data analysis consisted of descriptive statistics and frequency analysis (Chi^2^-Tests). Moreover, the differences between nursing staff in elderly care, in hospitals, in home care and nursing trainees were tested by using one-way Analysis of Variance followed by Post-Hoc Tests (Dunn–Bonferroni Test, Dunnett Test). Cohen’s *d* was calculated to analyze effect sizes between groups. Therefore, the data were tested for normal distribution. Normal distribution was not confirmed for the physical score. For this data we used the Kruskal–Wallis Test. Nominally and ordinally scaled data were analyzed by Chi^2^-Tests. All Analyses were performed with SPSS version 22 (IBM SPSS Statistics for Windows, Armonk, NY, USA).

## 3. Results

Table 1 describes the scores of the Physical and Mental Health and Chronic Stress. Data is presented as means ± standard deviations (SD).

Regarding the Mental Score no significant differences were found (F_(3217)_ = 1.943, *p* = 0.124), whereas the Trier Inventory for Chronic Stress resulted in significant differences in SSCS-values (F_(3236)_ = 5.775, *p* = 0.001). All groups showed SSCS-values above 15 and therefore were considered chronically stressed. Post-Hoc (Dunnett-Test) comparisons revealed a significant difference between nursing staff in elderly care and hospitals (*p* = 0.048). The comparisons of the means indicated small to large effect sizes (d = 0.024 to d = 0.757).

Highly significant differences were found in Physical Score (Chi^2^ = 24.734, *p* < 0.001). The subsequent Post-Hoc Test (Dunn–Bonferroni Test) showed that the groups nursing staff in elderly care and nursing staff in hospitals (z = –3.231, *p* = 0.007), nursing staff in elderly care and nursing staff in home care (z = –3.271, *p* = 0.006) as well as nursing staff in elderly care and nursing trainees (z = –3.707, *p* = 0.001) differ significantly. Significant differences between other groups were not noticed. However, the observed differences showed in Table 1 had effect sizes between d = 0.086 and d = 0.813.

Nurses strain was primarily characterized by:

- Heavy physical tasks (66.1% nursing staff in elderly care, 59.1% nursing staff in hospitals, 70.0% nursing staff in home care, 55.6% nursing trainees; Chi^2^ = 59.213, *p* < 0.001);
- Awkward posture (65.3% nursing staff in elderly care, 77.3% nursing staff in hospitals, 80.0% nursing staff in home care, 75.0% nursing trainees, Chi^2^ = 77.796, *p* < 0.001);
- Lifting heavy load (61.9% nursing staff in elderly care, 50.0% nursing staff in hospitals, 55.0% nursing staff in home care, 66.7% nursing trainees; Chi^2^ = 105.987, *p* < 0.001);
- Holding heavy load (51.3% nursing staff in elderly care, 52.3% nursing staff in hospitals, 60.0% nursing staff in home care, 44.4% nursing trainees; Chi^2^ = 89.762, *p* < 0.001);
- Time pressure (67.8% nursing staff in elderly care, 79.5% nursing staff in hospitals, 80.0% nursing staff in home care, 69.4% nursing trainees; Chi^2^ = 45.957, *p* < 0.001);
- Pressure to perform (55.9% nursing staff in elderly care, 70.5% nursing staff in hospitals, 60.0% nursing staff in home care, 66.7% nursing trainees; Chi^2^ = 62.872, *p* < 0.001).

**Figure 1:**
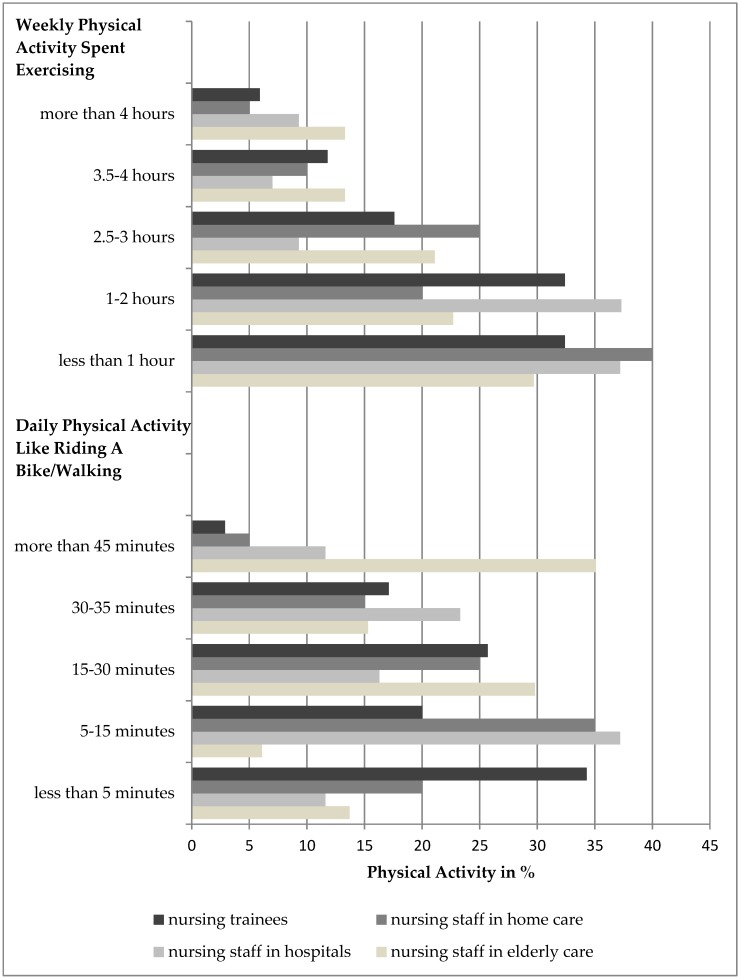
Daily and weekly physical activities.

Figure 1 illustrates daily physical activity in minutes, which included activities like riding a bike or walking and the number of hours per week spent exercising/playing sports. Significant differences were found between the types of nurses in daily physical activity (Nursing staff in elderly care; Chi^2^ = 54.992, *p* < 0.001).

**Figure 2:**
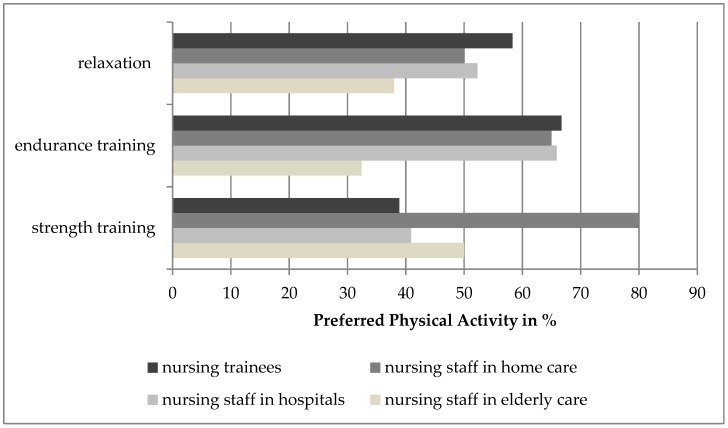
Preferred physical activities (multiple selections allowed).

Figure 2 shows the preferred physical activities for health promotion programs. Deviations were found in strength training (nursing staff in home care; Chi^2^ = 10.369, *p* = 0.016) as well as in endurance training (nursing trainees; Chi^2^ = 26.669, *p* < 0.001).

**Figure 3:**
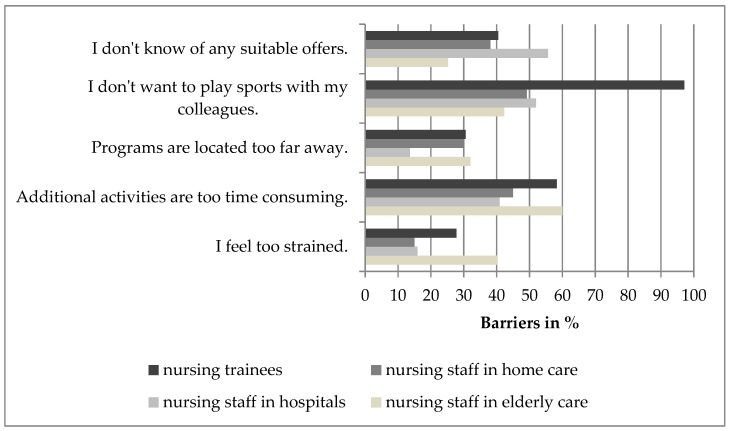
Barriers for participating in health promotion programs (multiple selections allowed).

In contrast to other nurses, 40.3% of nurses in elderly care indicate that they are too strained to attend any physical activity or other additional program (Chi^2^ = 12.364, *p* = 0.006). In addition, the time expenditure is too high for nearly half of the nurses. Also, nursing trainees in particular do not want to play sports with their colleagues (Chi^2^ = 23.602, *p* < 0.001) (Figure 3).

The control for gender differences did not show any effects.

## 4. Discussion

The aim of this cross-sectional study was to analyze whether nursing staff in elderly care, hospitals, home care, and nursing trainees show different levels of work-related burdens and whether they require tailored and individualized components in an exercise and health promotion program.

Highly significant differences were found in work-related burdens like Chronic Stress, Physical Health, and Nurse’s Strains, as well as daily physical activity and the requirements for an individualized health promotion program. 

In contrast to Nübling et al. [13], the average mental scores of nursing staff in different roles were similar and beneath the reference values of the German population (51.58 ± 8.05). This could be due to lack of support, little income, low social prestige, as well as high workloads and shift work of nurses. Simultaneously the survey participants showed average SSCS-values above 15 and therefore were considered chronically stressed, with nurses in home care showing the highest SSCS-values. These results confirm the findings of previous studies which also demonstrated higher stress levels among nurses than other professions [10,11]. The comparisons between the different roles regarding stress presents a significant difference between nursing staff in elderly care and hospitals as well as high effect sizes between nursing staff in elderly care and home care. As a frequent source of stress participants of all roles stated time pressure and pressure to perform, even though time pressure seemed to be the most straining in home care (80%). Time pressure and pressure to perform as well as high stress levels may be a consequence of high rates of multi morbidity and staff shortage [2,3]. Since nurses in home care usually work alone, higher frequencies of perceived time pressure and resulting stress seems plausible, even though these results are contrary to Nübling et al. [13], who found lower values in home care nurses for quantitative and emotional demands. In addition these results are contrary to Hasson and Arnetz [12], who reported significantly higher stress ratings in nursing homes compared to home care. Besides, these results are in accordance to Wenderlein [14], who showed higher emotional burdens in nursing trainees compared to nursing staff. Nursing trainees often need more time for optimal care of residents or patients and were therefore under increasing time pressure and pressure to perform. In addition, they felt burdened by unclear or contradictory work instructions [14]. As a consequence, nurses in all settings require specific health promotion programs focusing on stress management strategies, especially in regard to managing time pressure issues. 

In addition to psychological strains, physiological strains play a major role in care. Owen et al. [8] identified staff shortage as one factor that increased the frequency of lifts per nurse per shift due to the patient/resident-to-nurse ratio. In the present survey, the nurses stated heavy physical tasks, awkward postures, lifting, and holding heavy loads as physical demands. These may consequently be a result of current trends of work intensification in the German health sector, leading to an increase of patient or resident handling tasks such as heavy lifting, transferring, bending and twisting for each nurse [18]. In particular, nursing staff in home care were burdened by heavy physical tasks, awkward postures, and holding heavy loads, although they have the lowest lifting and bending exposure according to Simon et al. [20]. This could be due to the structural conditions of the private apartments of the patients and the lack of help from colleagues. The physical well-being of the nurses in elderly care differed significantly and were beneath the reference values, which suggest that the high demands affect the well-being. In spite of the high demands, nurses in home care surprisingly had the highest physical well-being and were thus above the reference values of the German population (50.22 ± 8.68). However, due to the small sample size of this group one could speculate if the combination of home care and driving from one patient to the next might reduce the time working in awkward body positions and allows more breaks in comparison to the nurses in elderly care. In future research, the resources of nurses in home care should be further investigated. The results therefore indicate that particularly nurses in home care and elderly care need to be addressed with ergonomic movement programs.

On the other hand, the self-reported physical activity revealed that nurses in elderly care were the most active in daily life and spent the most time a week exercising or playing sports. In this study, 43.7% of nurses in elderly care, 25.6% of nurses in hospitals, 40.9% of nurses in home care, and 35.3% of nursing trainees stated to spend more than 150 min a week exercising or playing sports. The WHO recommends at least 150 min of moderate-intensity aerobic physical activity throughout the week; hence a total of 36.4% of nurses in this study fulfilled WHO recommendations which is a higher percentage than among the average German population (22.6%) [47]. These results seem somewhat surprising considering previous studies which reported low physical activity levels among nursing personnel as well as nursing trainees [36,37]. It should, however, be noted that self-reported questionnaires assessing the level of physical activity have proven to be problematic [48,49]. The exercise per week stated by the caregivers might not fulfil the definition of moderate intensity of the WHO (“activity that is performed at 3.0–5.9 times the intensity of rest. On a scale relative to an individual’s personal capacity, moderate-intensity physical activity is usually a 5 or 6 on a scale of 0–10” (WHO, p.16) [50]. It could be speculated that the physical activity performed by the nurses in this study does not include physical activity that is beneficial to health or does not reach the required intensity and therefore has not shown the discussed impacts on mental health and perceived stress. In future research, this aspect must be considered. 

Regarding health promotion programs, nursing staff in elderly care and home care preferred strength training. Reasons for the preferred strength training in elderly and home care might be the increasing number of people in need of care who are immobile. Another reason might be the ergonomic conditions of private households such as missing nursing beds and constricted rooms. On the contrary, nurses in hospitals and trainees wished for endurance training presumably to compensate psychological strains.

Otherwise nurses did not want to spend time participating in additional activities (60% nurses in elderly care, 40.9% nurses in hospitals, 45% nurses in home care, and 58.3% nursing trainees). As a result, the nurses are unlikely to participate in additional activities outside working hours. The health promotion programs should therefore take place during working hours, especially for nurses in elderly care and nursing trainees. In addition, the nursing trainees in particular do not want to play sports with their colleagues (Chi^2^ = 23.602, *p* = 0.000). However, the barrier can also be found in nurses from the other settings. This suggests that they need individualized training, a training to break down this barrier or in particular in case of the trainees—a training which can be integrated in school life. Nursing staff in elderly care also feel too strained to participate in health promotion programs (Chi^2^ = 12.364, *p* = 0.006). The organization of the programs is therefore important considering the shortage of skilled workers. Float nurses could relieve the burden on nurses in elderly care to enable them to participate in programs. Furthermore, the lack of knowledge of suitable offers and the distance to the places where programs are conducted were the most mentioned barriers keeping the nurses in this study from participating in health promotion programs. Consequently, we recommend implementing health promotion programs during working time at the work setting. This might increase the motivation for participating because the nurses do not have to spend additional time and to change the location. Planning should be carried out on a long-term basis so that the health promotion program can be integrated into the working schedule. Also, the programs should be offered during shift change periods to enable more nurses to participate. Thus, it is also recommended that more coordination and communication between decision makers and planners in the area of the programs and nursing care management as well as residential management should take place in order to regulate working shifts. This is in line with Hamzehgardeshi and Shahhosseini [38], who reported that organizational barriers are the most important barriers for participation in programs in nursing settings. Overall, it is therefore particularly important to choose a bottom-up approach, to include the nursing staff in planning and to dismantle the barriers. Furthermore, programs must clarify the positive effect of such programs on the individuals health status.

Given the fact, that the nurses in different roles are highly burdened, tailored interventions are needed that take into account setting specific factors as well as wishes, needs and barriers of the different settings. According to Van Hoof et al. [51] and Edwards et al. [33] the stressors of nurses are well researched, but a translation of these results into well evaluated interventions is lacking. Further research should consider barriers and facilitators found in this study.

### Limitations

Nevertheless, our cross-sectional study has some limitations. Nursing staff were allowed to complete the survey during working time. It took approximately 30 min to complete the entire questionnaire. Lack of time and time pressure could have influenced the quality of the answers. Furthermore, the sample size of nurses in home care is smaller compared to that of the other groups. This might result in a small sampling bias. 

Moreover, the results were not controlled for the duration of employment. Working with the identified work-related stressors might increase burdens and strains over time. Therefore, this aspect should be considered in future studies. Another limitation could be the different age of the nurses. However, the fact that the nurses in elderly care were about ten years older merely reflects the situation of the labor market in nursing sector. Nevertheless, aging is an important factor for musculoskeletal problems that should be taken into account: Age-related factors should be considered when designing interventions, especially when addressing different target groups, such as trainees, younger, and older nursing staff. Finally, the survey did not integrate aspects of work-life-balance, especially time conflicts that diminish the recreation time of the nursing staff. Therefore, ongoing research on intervention planning should also address these time restrictions for individual health promotion. 

## 5. Conclusions

This study identified the work-related burdens and the requirements for health promotion programs in nursing settings. This is relevant for the development of effective and tailored interventions to reduce work-related burdens in nurses.

High chronic stress values, low mental health scores as well as high physical and mental strains in nurses of all roles indicate the importance of health promotion programs. Nurses in different settings need to be addressed with different health promotion strategies.

Contrary to earlier studies, nurses in home care in this study seem to have the most difficult working conditions and are therefore exposed to the highest physical strains and stress levels. The desired strength training should therefore be combined with ergonomic movement programs. In addition, a focus should also be placed on stress management strategies in regard to managing time pressure issues. When designing health promotion programs for home care personnel, it should be taken into account that home care nurses feel programs to be too time consuming and feel reluctant to doing physical activity in front of colleagues.

Overall, health promotion programs for all nursing settings should be implemented during working time and should consider the working schedule in order to facilitate participation. Nevertheless, different nursing settings need targeted programs. These findings have provided a foundation to counteract work-related burdens and design health promotion programs in different nursing settings accordingly.

## Figures and Tables

**Table 1:** Scores.

Scores	Nursing Staff in Elderly Care (n = 142)	Nursing Staff in Hospitals (n = 44)	Nursing Staff in Home Care (n = 20)	Nursing Trainees (n = 36)
**SF12-Health Survey**				
Physical Score	48.23 ± 9.80	53.31 ± 7.07	54.77 ± 5.76	54.20 ± 7.50
Mental Score	46.36 ± 10.33	43.72 ± 9.84	44.40 ± 12.21	41.85 ± 11.62
**Trier Inventory for Chronic Stress**				
Screening Scale Chronic Stress (SSCS)	17.82 ± 10.64	22.61 ± 10.08	26.10 ± 12.86	22.86 ± 10.65
